# Supplementary material for: Satellite Tracking of Head-Started Juvenile Green Turtles (Chelonia mydas) Reveals Release Effects and an Ontogenetic Shift
Source: Animals (Basel). 2023 Mar 31;13(7):1218. doi: 10.3390/ani13071218 (PMC10093175; doi:10.3390/ani13071218)
Supplement: Supplementary file 1 [file animals-13-01218-s001.zip › animals-2259595-supplementary.pdf]

Table S1. Individual-level (N=40) data for: age class, number of raw locations, body mass (kg), curved carapace length (CCL, cm) and width (CCW, cm), tag (S= Solar, NS= non-solar/satellite) and adhesive (E= epoxy, FC= Fast Cure) type, median timestep between raw locations (hrs), and tag duration (days). Individuals 214082 and 203086 are greyed out, as they only had one location each outside the boundaries of Grand Cayman Island and were removed from further analysis.

ID	Tracking Period	Movement Pattern	Age Class	No. of Raw Locations	Mean Locations *per day*	Body Mass *kg*	CCL *cm*	CCW *cm*	Adhesive Type	Tag Type	Median Timestep *hrs*	Days Tracked *days*
**202985**	Jan	Residential	1-2 Yrs	15	1.6	5.3	37	32	E	NS	22.7	10.0
**203084**	Jan	Dispersive	1-2 Yrs	32	3.7	4.3	37	29	FC	NS	11.9	23.5
**203407**	Jan	Residential	1-2 Yrs	79	6.6	5.6	37	32	FC	S	10.5	12.5
**203408**	Jan	Dispersive	1-2 Yrs	40	5.8	4.2	35	30	E	S	11.3	18.0
**203410**	Jan	Dispersive	1-2 Yrs	126	14.2	4.3	35	31	FC	S	1.7	12.5
**212846**	Jan	Residential	1-2 Yrs	22	3.7	4.3	35	31	E	NS	10.8	32.5
**214069**	Jan	Dispersive	1-2 Yrs	19	3.5	6.8	40	34	FC	NS	12.0	10.5
**214083**	Jan	Residential	1-2 Yrs	87	4.1	4.8	38	31	FC	S	11.0	29.5
**214085**	Jan	Dispersive	1-2 Yrs	115	12.7	5.0	38	33	E	S	2.4	16.5
**203436**	Jan	Residential	1-2 Yrs	5	1.0	6.3	40	31	E	NS	17.7	3.0
**203080**	Jan	Residential	2-3 Yrs	9	2.0	12.8	51	43	E	NS	23.3	10.0
**203087**	Jan	Dispersive	2-3 Yrs	28	2.6	17.2	53	46	E	NS	11.2	12.5
**203403**	Jan	Residential	2-3 Yrs	50	4.2	23.3	61	51	E	S	11.2	11.5
**203404**	Jan	Residential	2-3 Yrs	13	2.5	12.9	51	42	E	S	13.2	5.4
**203406**	Jan	Residential	2-3 Yrs	16	3.6	19.5	62	51	E	S	6.1	3.5
**212847**	Jan	Dispersive	2-3 Yrs	37	3.5	14.8	51	46	E	NS	12.1	18.9
**212867**	Jan	Dispersive	2-3 Yrs	29	2.8	14.2	51	44	E	NS	11.3	23.0
**214081**	Jan	Dispersive	2-3 Yrs	60	7.4	9.6	44	38	E	S	10.7	10.5
214082	Jan	-	2-3 Yrs	1	-	11.6	47	41	E	S	-	0
203086	Jan	-	2-3 Yrs	1	-	15.2	51	45	E	NS	-	0
**203089**	Jan	Residential	3-4 Yrs	13	1.4	22.7	60	51	E	NS	22.3	19.0
**203405**	Jan	Residential	3-4 Yrs	27	9.1	22.6	59	52	E	S	1.3	3.0
**203409**	Jan	Dispersive	3-4 Yrs	41	3.0	29.4	65	55	E	S	12.5	15.0
**203411**	Jan	Dispersive	3-4 Yrs	207	7.8	20.4	58	51	E	S	1.8	28.1
**203412**	Jan	Residential	3-4 Yrs	6	2.6	24.9	58	53	E	S	12.0	3.0
**212837**	Jan	Residential	3-4 Yrs	26	2.9	29.5	62	54	E	NS	10.7	11.5
**212845**	Jan	Dispersive	3-4 Yrs	10	2.2	29.4	62	52	E	NS	23.7	5.6
**212862**	Jan	Residential	3-4 Yrs	25	1.9	20.4	54	48	E	NS	23.3	19.0
**212866**	Jan	Residential	3-4 Yrs	13	2.8	13.6	54	44	E	NS	47.8	19.0
**228045**	Jan	Residential	3-4 Yrs	73	8.3	31.8	62	53	E	S	10.7	18.5
**229669**	Jul	Dispersive	1-2 Yrs	55	13.3	5.1	37	32	FC	S	1.3	11.6
**229670**	Jul	Dispersive	1-2 Yrs	56	12.3	5.7	38	33	FC	S	1.1	24.6
**229671**	Jul	Dispersive	1-2 Yrs	60	16.1	6.3	40	34	FC	S	0.9	10.5
**229672**	Jul	Dispersive	1-2 Yrs	43	12.6	5.2	39	32	FC	S	1.5	9.5
**229673**	Jul	Dispersive	1-2 Yrs	54	14.8	5.3	37	33	FC	S	1.2	10.5
**229674**	Jul	Dispersive	1-2 Yrs	42	10.1	5.0	37	32	FC	S	1.3	10.5
**229675**	Jul	Dispersive	1-2 Yrs	59	17.1	6.4	38	34	FC	S	0.8	10.6

**229676**	Jul	Dispersive	1-2 Yrs	55	17.4	4.9	36	32	FC	S	1.1	10.6
**229677**	Jul	Dispersive	1-2 Yrs	54	12.7	5.3	36	33	FC	S	1.2	10.5
**229678**	Jul	Dispersive	1-2 Yrs	41	15.4	4.8	36	33	FC	S	1.0	8.0

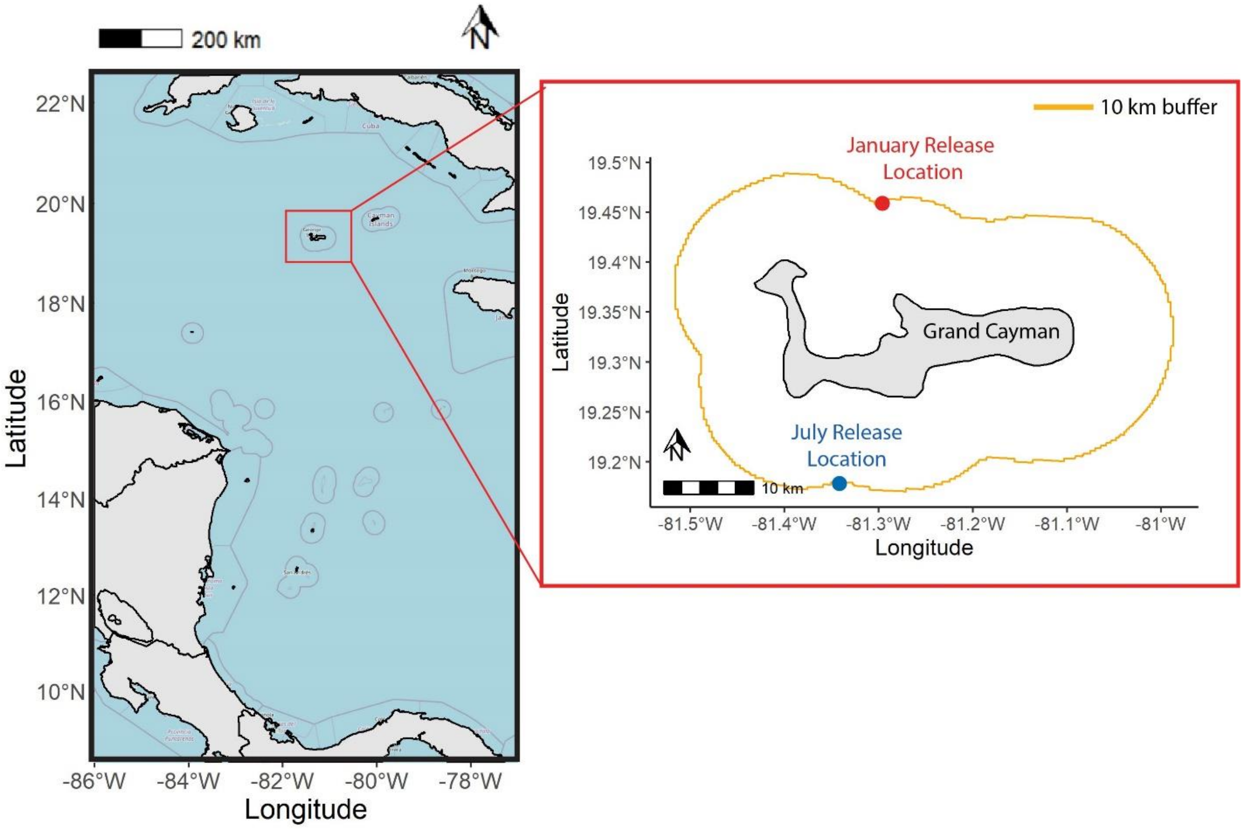

Figure S1. Map of the study region, with the red box showing a zoomed up view of Grand Cayman island (the captive rearing location) of the Cayman Islands, the January (red) and July (blue) release locations, and a 10-km buffer around the Island (yellow), which was used for filtering and data processing (see *Methods*).

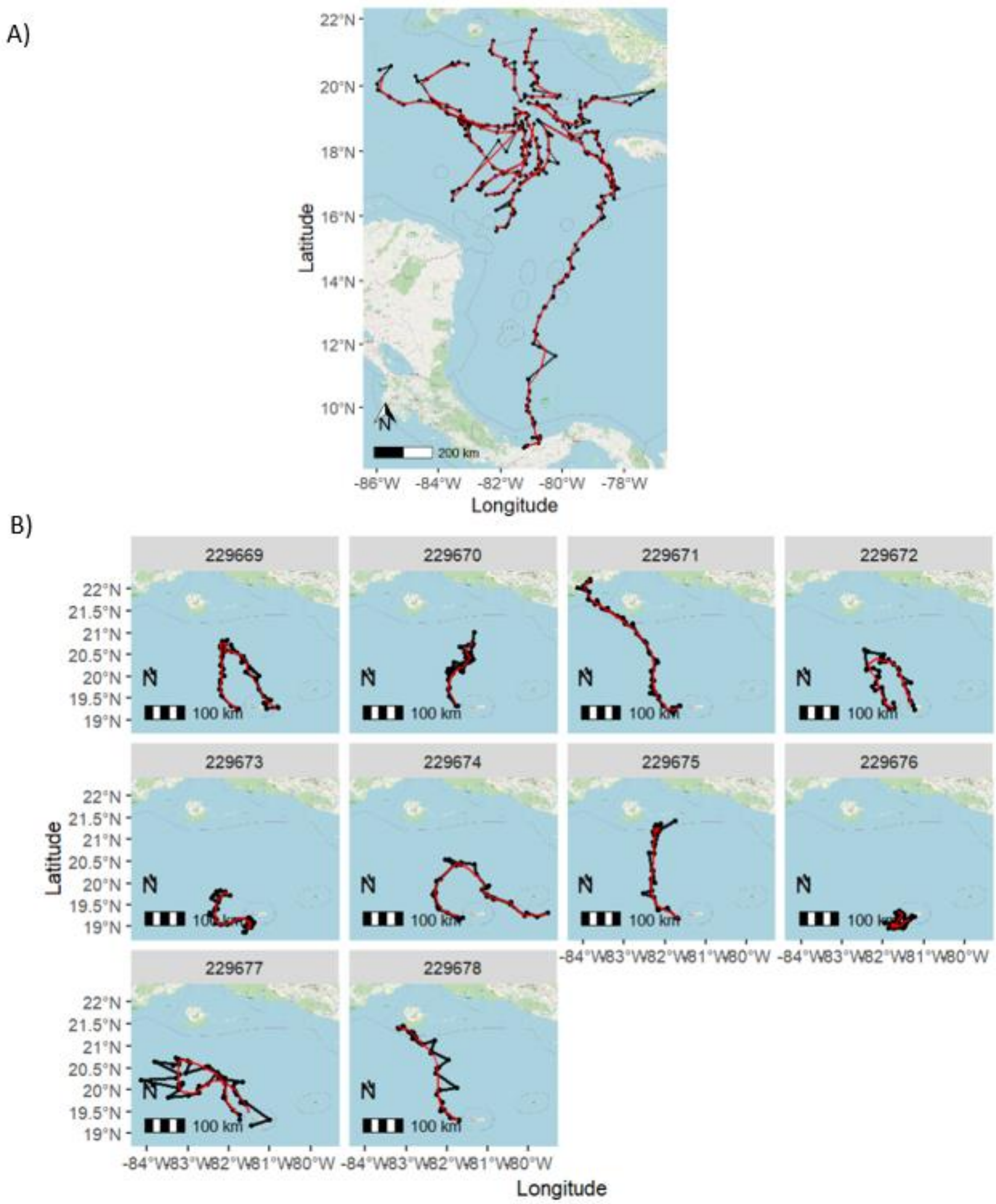

Figure S2. Maps of raw tracks and positions (black) overlaid with interpolated trajectories (red) for dispersive individuals in A) the January tracking period (N=12) and B) the July tracking period (N=10). To match the resolution of environmental data products and filter out error positions, locations within a 1 km buffer of the island were removed prior to interpolation. All remaining raw locations were filtered and regularized through the "foieGras" R package (Jonsen et al. 2019), resulting in daily positions for each individual.

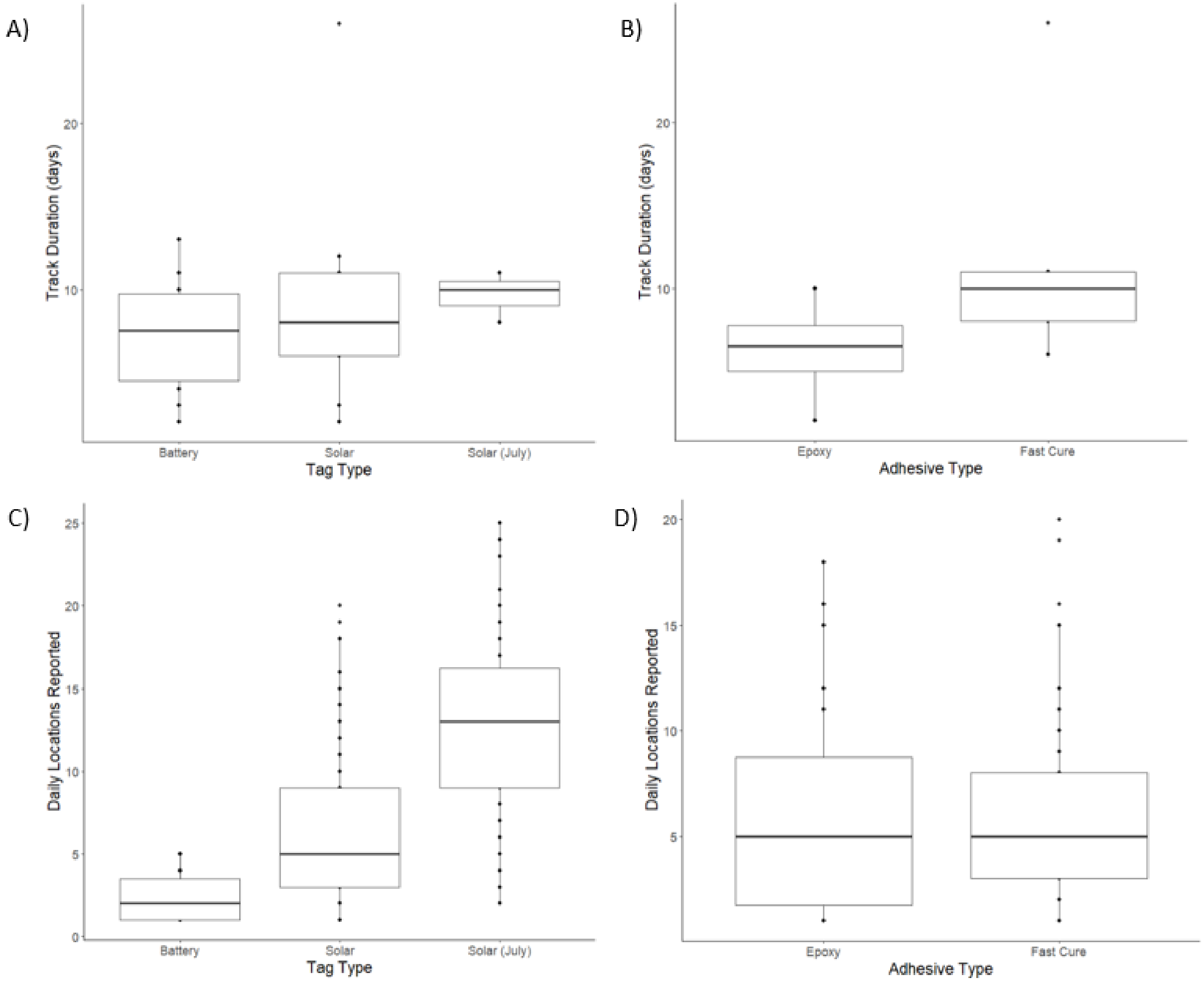

Figure S3. Boxplots of the tag duration by A) tag type (battery-powered vs. solar satellite tags) for all N=40 turtles and B) attachment type (fast-cure vs. epoxy) for N=10 turtles in the 1-2 year age class released in January. Note that turtles released in July are shown in a separate group due to being released at a different time period and location. Boxplots are also shown for the daily number of locations reported for C) tag type (N=40) and D) attachment type (N=10 turtles in the 1-2 years age class, released in January).

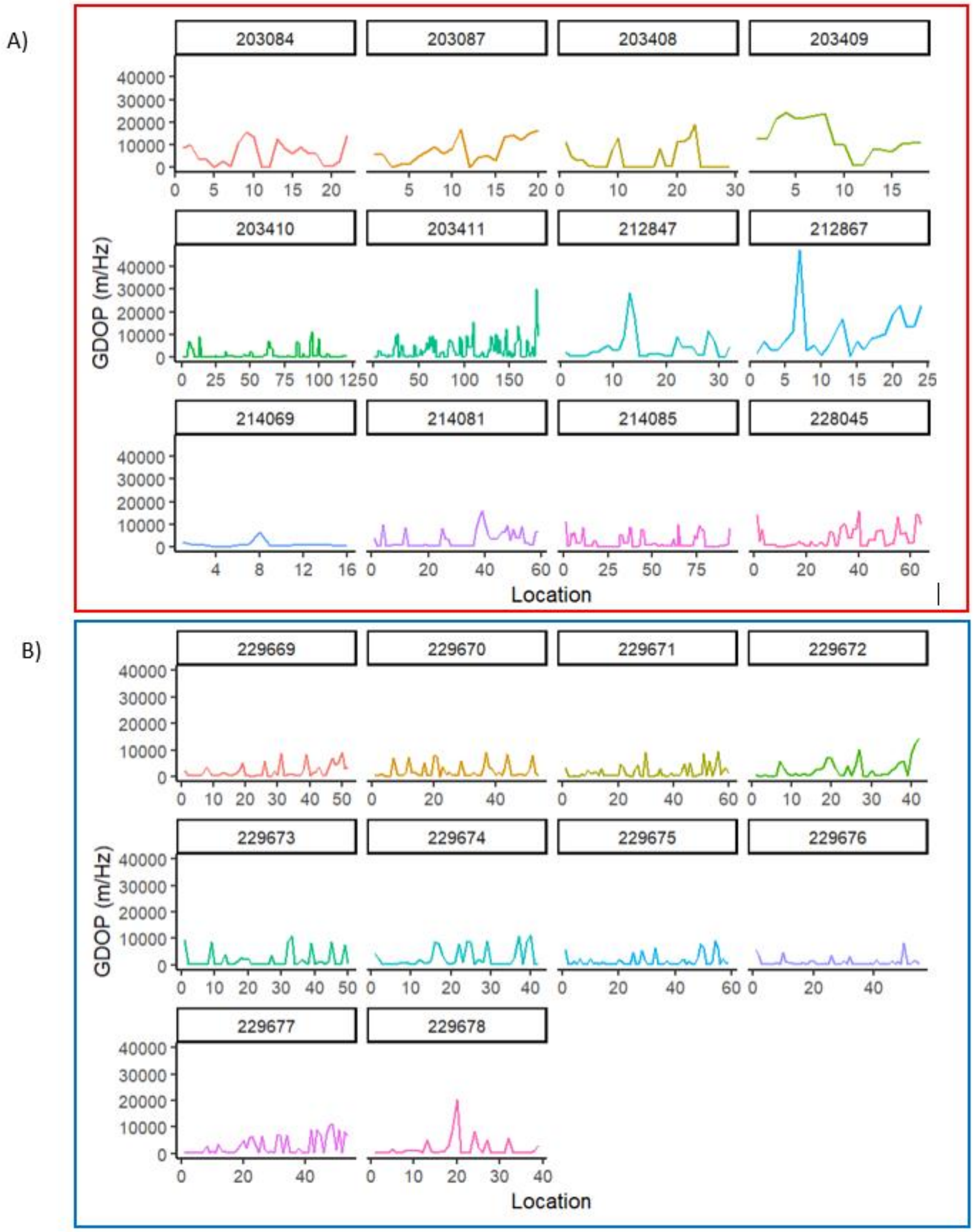

Figure S4. Plots of the Geometric Dilution of Precision (GDOP, a measure of ARGOS location accuracy error) values as a function of raw location number for A) January and B) July turtles.
